# Prevalence of Paid Sex and Associated Factors Among Women and Men Attending HIV Voluntary Counseling and Testing in Kinshasa, Democratic Republic of the Congo: A Prospective Cohort

**DOI:** 10.1007/s10508-024-02939-w

**Published:** 2024-08-15

**Authors:** Silvia Carlos, Gabriel Reina, Eduardo Burgueño, Benit Makonda, Jokin de Irala, Carlos Beltramo, Paula Díaz Herráez, Cristina Lopez-del Burgo

**Affiliations:** 1 Preventive Medicine and Public Health Department, University of Navarra, Pamplona, Spain; 2 IdiSNA, Navarra Institute for Health Research, Pamplona, Spain; 3 Institute for Culture and Society, University of Navarra, Pamplona, Spain; 4 Infectious Diseases and Microbiology Department, Clínica Universidad de Navarra, Pio XII Av, 36, 31008 Pamplona, Spain; 5 Soins Primaires Monkole, Kinshasa, Democratic Republic of Congo; 6 Faculté de Médecine, Université Officielle de Mbujimayi, Kinshasa, Democratic Republic of the Congo; 7 CEFA-Monkole, Kinshasa, Democratic Republic of the Congo; 8 School of Medicine, University of Navarra, Pamplona, Spain

**Keywords:** HIV, Voluntary counseling and testing, Paid sex, Africa

## Abstract

Paid sex is associated with HIV and other sexually transmitted infections, which are highly prevalent in Sub-Saharan Africa (SSA). However, few data exist on this sexual practice among the general population in SSA, including the Democratic Republic of the Congo, where data on paid sex mainly comes from sex workers. In the DRC, most HIV Voluntary Counseling and Testing (VCT) centers do not discuss paid sex as a risk factor. Thus, we aimed to analyze the prevalence of paid sex, its associated factors and association with HIV among women and men attending HIV VCT at a reference hospital in Kinshasa. From 2016 to 2018, the Observational Kinshasa AIDS Initiative cohort analyzed the impact of HIV VCT on changes in HIV knowledge, attitudes, and sexual behaviors at follow-up. Participants aged 15–69 years were HIV tested and interviewed at baseline and at 6- and 12-month follow-ups. At baseline, participants were asked about their history of “ever” having had exchanged sex for money. At both follow-ups, the frequency of this practice was referred to as “the previous 6 months.” Descriptive, bivariate, and multivariate logistic regression analyses were carried out to evaluate the prevalence of paid sex, its associated factors, and the association between paid sex and HIV. Statistical analyses were performed with Stata 15.1. Among 797 participants at baseline, 10% of those sexually experienced reported having ever had paid sex (18% men and 4% women, *p* < 0.001). At 6 and 12-month follow-ups, 5% and 2%, respectively. Paid sex was significantly and independently associated with being male (aOR = 2.7; 95% CI = 1.4–5.2), working or studying (aOR = 2.8; 95% CI = 1.5–5.0), daily newspaper reading (aOR = 4.4; 95% CI = 1.7–11.2); daily/weekly alcohol consumption (aOR = 3.3; 95% CI = 1.8–6.1), first sexual intercourse before age 15 years (aOR = 2.3; 95% CI = 1.1–5.0), multiple sexual partners (aOR = 4.1; 95% CI = 2.2–7.7), and extragenital sexual practices (aOR = 2.4; 95% CI = 1.3–4.4). A high religiosity (daily/weekly church attendance and praying) was inversely associated with paid sex (aOR = 0.1; 95% CI = 0.0–0.4). The high prevalence of paid sex among people attending HIV VCT in Kinshasa, associated with other sexual and consumption risk behaviors, highlights the need to include paid sex among the risk factors mentioned in HIV prevention counseling.

**Supplementary Information:**

The online version contains supplementary material available at 10.1007/s10508-024-02939-w.

## Introduction

Currently, the human immunodeficiency virus (HIV) and other sexually transmitted infections (STIs) are considered an important public health problem in Sub-Saharan Africa (SSA). The last updated data from UNAIDS estimates show that in 2021 60% of the new HIV infections worldwide take place in this region, where 25.6 million infected people live. In Eastern and Southern Africa a median HIV prevalence of 6.2% has been estimated among adults (aged 15–49 years), with a much higher prevalence among key populations, such as sex workers (33.4%); in Western and Central Africa the median prevalence is 1.3 and 8.9% among sex workers (UNAIDS, 2022a). Additionally, SSA has the highest prevalence of the four curable STIs (*T. vaginalis*, *C. trachomatis*, *N. gonorrhoeae,* and *T. pallidum*) (WHO, 2021; Zhang et al., 2022), as well as HPV (Bruni et al., 2021) or HSV-2 (James et al., 2020; Looker et al., 2020; Silva et al., 2022), among others.

HIV and STIs are associated with the engagement in paid or transactional sex (in which sex is exchanged for money or gifts) (Foley et al., 2013; Kiyingi et al., 2022; Maher et al., 2020; McMillan et al., 2018; Ruangtragool et al., 2022; Seidu et al., 2020; Stoebenau et al., 2023; UNAIDS, 2018; Wamoyi et al., 2019).

Data on the prevalence of this sexual practice in SSA mainly come from female sex workers. Much less information exists on the prevalence of purchase or sale of sex among the general population, which can be an important bridging group for HIV/STIs transmission to the rest of the community (Adal, 2019; Baltazar et al., 2021; Chatsika et al., 2020; Döring et al., 2022; Maher et al., 2020; Mantell et al., 2022; Ruangtragool et al., 2022; Seidu et al., 2019; Willis, 2013). A systematic review carried out in 2018 evaluated in nearly half of the SSA countries the prevalence of sex trade among youth (12–26 years) of the general population excluding people from high-risk populations. Among young males the prevalence of having ever bought sex ranged from 14 to 60% and it was 7 to 12% for selling sex. Among female youth information on buying sex was scarce, but a prevalence around 7% was reported and the lifetime prevalence of sold sex ranged from 5 to 85% (Krisch et al., 2019). There are less data on paid sex among adults from the general population. A recent meta-analysis including data from 35 African national population-based surveys, found that 8% of sexually active men reported having ever paid for sex, with a 68% prevalence of condom use at last paid sex in those surveys carried out since 2010 (Hodgins et al., 2022).

In the Democratic Republic of the Congo (DRC), it is estimated that around 0.8% of the adult population in the country are sex workers (UNAIDS, 2022a). The HIV prevalence among them is 7.5%, compared with 0.6% in the general population (UNAIDS, 2022). Regarding sex exchange with sex workers, a study focused on HPV infection among women in Kinshasa showed that 5 and 3% reported that their partners visited sex workers occasionally or frequently, respectively, before their current union (Sangwa-Lugoma et al., 2011). The 2013–14 National Demographic Health Survey (DHS) collected data among men aged 15–49 years and found that 28% of men reported having ever paid someone for sex and 11% in the previous year. In Kinshasa, the capital city, the prevalence was 23 and 8%, respectively. No data on women are available nor information on the associated factors (MPSMRM et al., 2014).

**Fig. 1:**
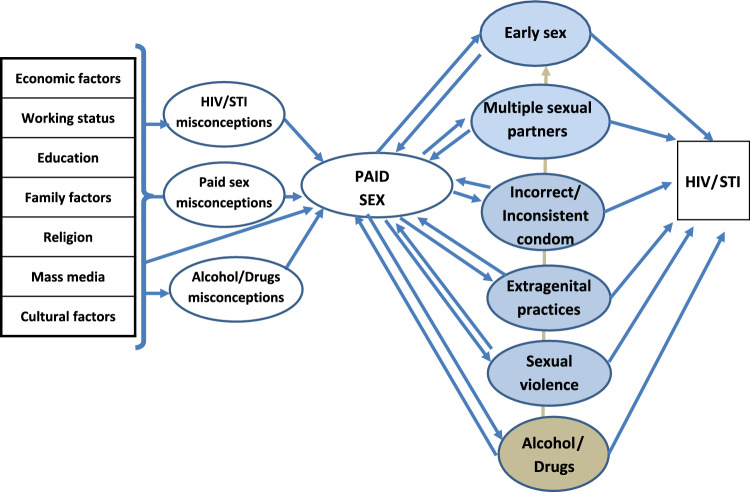
A theoretical framework for the determinants of paid sex in SSA Africa, based on the Knowledge-Attitudes-Practice (KAP) model

Paid sex often implies other risk behaviors associated with HIV/STI, such as early sex, multiple sexual partners, inconsistent or incorrect condom use, sexual violence or alcohol consumption (Ahinkorah et al., 2020; Kayembe et al., 2008; Longo et al., 2017; Mayanja et al., 2023; Ntumbanzondo et al., 2006; Ortblad et al., 2019; Stoebenau et al., 2023; Wamoyi et al., 2019; Yang et al., 2020). Behind all these risk behaviors, there are HIV/STI-related misconceptions and attitudes which are, in turn, determined by multiple structural, socioeconomic, and cultural factors (Green & Ruark, 2011; Mihretie et al., 2023) (Fig. 1).

The DRC is one of the poorest countries in the world, characterized by persistent sociopolitical instability. Within this context, sex work has become a common way to earn money, mainly among women and young girls (Apasa et al., 2018). However, there is often under-reporting of transactional sex as a result of the associated stigma, and additionally, valid and reliable data on this practice is often not registered in clinical practice or counseling guidelines and protocols (Kyegombe et al., 2021; Wamoyi et al., 2019; Steen et al., 2015; USAID, 2015). Paid sex is not usually discussed in HIV Voluntary Counseling and Testing (VCT) centers within the National HIV/AIDS Program of the DRC (PNMLS, 2020). Thus, from a public health point of view, and considering the complex nature of factors related to paid sex, it is very important to detect early in the community the prevalence of engagement in paid sex, as well as its determinants, in order to provide timely information about its possible health risks and prevention.

Considering that there are scarce data on paid sex in Kinshasa and on the associated factors determining this sexual practice, we aimed to (1) analyze the prevalence of paid sex among women and men attending HIV VCT at a reference hospital within the public healthcare system in Kinshasa, DRC; (2) evaluate the associated behaviors, misconceptions, and structural and sociocultural factors; and (3) analyze the association between paid sex and HIV.

## Method

### Participants

From April 2016 to April 2018, people aged 15–69 years attending HIV VCT at Monkole Hospital in Kinshasa were offered to participate in the Observational Kinshasa AIDS Initiative (OKAPI) prospective cohort study. As previously described, this study analyses the impact of HIV VCT on changes in HIV knowledge, attitudes, and sexual behaviors after a 6- and 12-month follow-up period (Carlos et al., 2021). At baseline, people with a previous HIV-positive test as well as pregnant women were excluded.

People attending VCT at Monkole Hospital are representative of the general population of the Mont-Ngafula area, where the hospital is located, and other surrounding areas (i.e., Ngaba, Selembao, or Lemba). It is a semi-urban area in the outskirts of Kinshasa, near some university campuses in Kinshasa. Specific high-risk groups are not known to be particularly present in these communities. VCT participants are not reached for HIV screening and there are no specific sensibilization campaigns for high-risk groups in the area.

Participants’ transportation to Monkole Hospital was paid on arrival to facilitate follow-up but no incentives were given for participation.

### Procedure

#### HIV Tests and Other STI Diagnoses

A blood sample was collected from each participant through venipuncture. Rapid diagnostic tests were used for HIV diagnosis, consistent with the local practices: first, a Determine® HIV-1/2 test was done and if it was positive, a Double Check Gold® and Unigold® rapid immunoassays were carried out. A dried blood spot (DBS) sample was also collected for additional external HIV analyses (i.e., subtyping and resistance analyses) and STI diagnosis. Additionally, participants self-reported if they had ever received an STI diagnosis.

#### Personal Interviews

After the participants accepted to in the study and were HIV tested, face-to-face personal interviews were held. Considering the high frequency of illiteracy in Kinshasa, local male and female interviewers were available to collect participants’ data, mainly in French and in Lingala in a few cases. They were local nurses highly sensitized to HIV and sexual health as they were working in VCT at Monkole Hospital during the study time. All baseline and 6- and 12-month follow-up interviews took place in a private room.

#### Questionnaire

Interviewers used a pen and paper questionnaire to collect data. Sociodemographic data as well as information about HIV knowledge, beliefs, attitudes, behaviors, and exposure to community HIV information were collected (Appendix). The questionnaires were built *ad-hoc* for the OKAPI project, based on previous projects at Monkole Hospital (Carlos et al., 2015, 2016) and on previously validated surveys, including the HIV-Knowledge-27-Scale, designed specifically for the sub-Saharan African population (Ciampa et al., 2012). The questionnaires included mainly closed questions (often on a Likert scale). They were initially designed in Spanish and translated by back-translation into Congolese French. The duration of the questionnaire implementation at baseline and follow-up surveys was about 35 and 20 min, respectively.

### Measures

Following the theoretical framework of the study (Fig. 1), the prevalence of the outcome variable, paid sex, was first evaluated, as well as its associated risk behaviors. Secondly, misconceptions and their sociodemographic and other structural determinants were analyzed.

#### Paid Sex

Information about paid sex was collected for both male and female participants. At baseline, participants were asked whether they had “ever” had any kind of paid sex (this could include purchase or sale). In both 6- and 12-month follow-up questionnaires, this question referred to the previous 6-month period. In all questionnaires, the possible answers were: “never,” “seldom,” “frequently,” and “I don’t want to answer.”

#### Alcohol Consumption and Sexual Risk Behaviors

Participants were asked about alcohol consumption in a normal week (“How often do you consume an alcoholic drink (beer, wine, whisky) in a normal week?”) with possible answers going from “never” to “more than twice daily.”

Data on different sexual behaviors frequent in Kinshasa, as shown in previous results from the OKAPI cohort as well as other previous studies at Monkole Hospital (Carlos et al., 2017, 2019), were collected: age at first sexual intercourse, multiple sexual partners, condom use and intention of use, sexual violence, and oral and anal sex.

#### HIV Misconceptions

Misconceptions about HIV and its transmission are highly prevalent in Kinshasa, as previously shown by this research group (Carlos et al., 2015); thus, this variable was collected in the OKAPI cohort questionnaire, including believing that someone HIV+ cannot look healthy, HIV is a punishment from God, and HIV is transmitted by sorcery, a kiss on the mouth or mosquito bites.

#### Sociodemographic Factors

Data on different sociodemographic characteristics were collected, including sex, age, education, economic level, professional status, religion and religiosity (church attendance and praying), and media access.

### Statistical Analysis

#### Sample Size Calculation

Considering a *z* = 1.96 (for a 95% CI), an estimated paid sex prevalence of 23% (DHS, 2014), and an error margin of 0.03, we estimated a sample size of 756 participants, following the formula *n* = *z*^2^*p*(1 - *p*)/error^2^. We estimated that this sample size would allow us to include enough parameters to analyze our objectives, considering that to perform multivariate analyses about 10 people are needed for each quantitative variable/indicator of qualitative variable in the model (Hosmer & Lemeshow, 2013).

Before performing the statistical analyses, data cleaning, errors correction and consistency checking were carried out. The presence of missing data was also evaluated, being its prevalence very low.

A descriptive analysis was first carried out to evaluate the baseline and 6- and 12-month follow-up prevalence of paid sex, as well as participants’ sociodemographic characteristics, HIV-related knowledge and perceptions, and other behaviors. All these descriptive analyses were further stratified by sex.

For all analyses concerning paid sex, a new dichotomous variable was created, “having had paid sex” (“ever” or “in the previous 6 months” if it was paid sex reported at baseline or follow-ups, respectively). The categories ‘seldom’ and “frequently” in the original variable were collapsed and classified as “having had paid sex” (“yes”) and those participants answering “never” were classified as “no” paid sex.

All categorical variables were described as percentages. The normal distribution of the quantitative variables was analyzed using Shapiro–Wilk test and the median and interquartile range (IQR) were calculated for those variables not following a normal distribution. The prevalence of paid sex and participants’ characteristics were compared between women and men using χ^2^ or Fisher exact tests for categorical variables and Student t tests for quantitative variables.

After the initial descriptive analyses, crude logistic regression analyses were carried out to evaluate, among participants sexually experienced, the factors associated with reporting paid sex. Afterward, multivariate regression models were adjusted in order to control for any confounding. Only the significant variables in the crude logistic regressions were kept to be included in the subsequent multivariate models. A first cross-sectional analysis was carried out to evaluate the association between baseline factors and reporting “ever having had paid sex” at baseline. The association between baseline and some follow-up variables and paid sex reported at 6- or 12-month follow-up was then analyzed. An additional multivariate analysis was carried out to evaluate the association between paid sex and having an incident HIV-positive test.

All analyses were carried out with STATA version 15.1 (StataCorp, College Station, TX, USA). All *p*-values < .05 were considered statistically significant.

#### Pilot Study

A pilot study was carried out before the beginning of the OKAPI project to test the questionnaire comprehension, measure the interview time, and check all the study protocols were appropriate. Some minor changes were made after the pilot study.

## Results

All people invited to participate agreed to join the study (100% response rate). At baseline, 797 participants replied to the baseline interview and were HIV tested. As previously described, at 6- and 12-month follow-up, retention rates were 57% (*N* = 456) and 27% (*N* = 219), respectively (Carlos et al., 2021).

### Participants’ Sociodemographic Characteristics

At baseline, 58% of the study population were women (Table 1). The median age of the participants was 28 (IQR: 12) years. Most participants had a middle economic level. Among those who reported their professional status, nearly 80% were studying or working. Regarding the education level, 56% had not completed their degree but nearly 70% of participants reported attending university. As expected, this percentage of incomplete university studies was significantly higher among young participants (84%) than among adults (42%). Around half of the participants reported daily access to the Internet and over 90% daily use of their mobile phones. Regarding their religion, 42% were Christians (Catholics and protestants) and 51% belonged to *Église de réveil*. Overall, the vast majority had a high religiosity. The majority of participants had a partner at study time and lived with their partner(s) but only 18% of the respondents reported being married (a higher proportion among men).

**Table 1:** Baseline characteristics of OKAPI cohort participants interviewed at different study times

	Baseline (*N* = 797)		6-month follow-up (*N* = 456)		12-month follow-up (*N* = 219)	
Baseline characteristics	Women (*n* = 462) %	Men (*n* = 335) %	Women (*n* = 269) %	Men (*n* = 187) %	Women (*n* = 121) %	Men (*n* = 98) %
*Sociodemographics*						
Young (15–24 yrs) (*n* = 258)	43.3	17.3	39.4*	16.0*	32.2*	9.2*
*Economic level*						
Low (*n* = 149)	15.8	22.7	18.2*	24.1*	14.1	29.6*
Middle (*n* = 617)	79.6	74.3	78.8	73.3	83.5	67.3
High (*n* = 31)	4.6	3.0	3.0	2.7	2.5	3.1
University studies (*n* = 531)	63.2	71.3	63.9	73.3	66.9*	75.5*
*Professional status*						
Unemployed (*n* = 102)	31.3	15.2	57.2*	43.3*	59.5*	44.9*
Student (*n* = 111)	32.6	18.3	21.6	13.9	21.5	14.3
Working (*n* = 211)	36.1	66.5	21.2	42.8	19.0	40.8
*Media access (daily)*						
Radio (*n* = 153)	10.0	31.9	11.5	33.2	9.1	35.7
Television (*n* = 369)	47.4	44.8	51.7	47.1	51.2	48.0
Newspaper (*n* = 42)	3.9	7.2	4.1	8.6	2.5	10.2
Internet (*n* = 388)	46.7	51.3	49.1*	54.5*	55.4*	57.1*
Mobile phone (*n* = 740)	91.1	95.2	92.6	94.1	92.6	98.0
*Religion*						
Catholic (*n* = 116)	22.3	33.5	23.9	30.9	23.2	36.9
Protestant (*n* = 64)	13.3	17.3	13.8	21.1	17.4	21.5
*Église de réveil (n* = *216)*	58.8	41.4	59.3	38.2	55.1	55.1
Weekly/daily church attendance/praying (*n* = 724)	95.5	84.5	95.9	84.0	93.4	83.7
Married (*n* = 147)	12.4	26.9	12.3	26.7	9.1	26.5
Living with partner (*n* = 666)	81.6	86.3	79.2	84.0	84.3	86.7
HIV misconceptions	23.6	18.5	23.4	17.1	14.0	14.3
*Risk Behaviors*						
Alcohol consumption (daily/weekly)(*n* = 135)	11.5	24.5	8.9	20.9	12.4	21.4
First sexual intercourse before 15 years (*n* = 81)	11.6	10.5	9.4	9.8	14.8	8.9
≥ 2 current partners (*n* = 134)	13.1	27.5	14.6*	26.8	17.6*	24.4
≥ 2 partners in last 6 months (*n* = 99)	7.8	18.8	8.2*	17.1	10.8*	17.4
Concurrent partners in last 6 months (*n* = 270)	24.2	47.2	23.1	47.1	27.3	45.9
*Condom use*						
Never (*n* = 115)	19.5	10.9	16.3*	11.5	11.2*	9.9
Sometimes (*n* = 593)	78.6	85.3	82.1	86.2	86.2	85.7
Always (*n* = 20)	1.9	3.8	1.6	2.3	2.6	4.4
*Extragenital practices*						
Oral sex (*n* = 432)	58.8	60.1	58.9	58.1	59.5	58.2
Anal sex (*n* = 160)	21.0	23.3	24.4	21.3	19.0	16.5
Sexual violence (*n* = 96)	16.9	8.3	6.7	6.4	6.0	7.0
*HIV test*						
Negative (*n* = 747)	94.8	92.2	95.5	92.0	95.9	93.9
Positive (*n* = 24)	4.1	1.5	3.7	1.1	3.3	1.0
Undetermined (*n* = 26)	1.1	6.3	0.7	6.9	0.8	5.1
Previous HIV test (*n* = 509)	64.9	62.4	67.7*	67.9*	69.4*	72.4*
Some HIV risk perceived (*n* = 151)	16.5	22.4	17.1*	20.9	20.7*	23.5
STI diagnosis in previous year (*n* = 80)	12.1	7.2	13.4	6.4	10.7	8.2

*STI* sexually transmitted infection

*Data show the characteristics of the study population that changed from baseline to follow-up

### Prevalence of Paid Sex

Regarding paid sex, among 728 participants sexually experienced at baseline, 10% (*n* = 73) reported having ever had paid sex. The prevalence was significantly higher among men (18%) compared to women (4%) (*p* < .001). Only 6 out of the 728 participants (0.75%) refused to answer the question about transactional sex.

At 6-month follow-up, the prevalence of paid sex in the previous 6-month period was 5% (*n* = 16) (8% among men and 3% among women, *p* = .04); 77% of them had also reported paid sex at baseline. At 12-month follow-up, only 2% (*n* = 3) reported paid sex in the previous 6 months (2 and 1% among men and women, respectively, *p* = .66). Among those participants reporting having ever had paid sex at baseline, 18% said they had it “frequently.” For those reporting follow-up paid sex in the previous 6 months, 13 and 0% reported frequent paid sex at 6- and 12-month follow-ups, respectively.

Among respondents ever having paid sex, nobody reported consistent condom use and 10% said they would never use a condom if they happened to have sex with a sex worker in the future.

### Prevalence of Other Sexual and Consumption Risk Behaviors

Both men and women reported a similar frequency of sex under 15 years (11%) and a very high frequency of oral sex (59%) (Table 1). Men were significantly more likely to report multiple serial and concurrent sexual partners and higher condom use.

As shown in Table 1, one out of four men reported daily or weekly alcohol drinking, which was two times significantly more frequent than among women’s consumption (*p* < 0.001).

### HIV-Related Misconceptions

Around 20% of the participants had HIV-related misconceptions and wrongly believed HIV is caused by witchcraft or God’s punishment.

### Factors Associated with Paid Sex

**Fig. 2:**
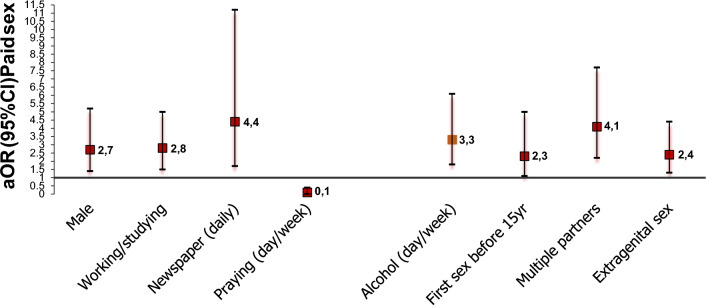
Factors associated with paid sex

At baseline, reporting paid sex was significantly and independently associated with being male (adjusted OR = 2.7; 95%CI = 1.4–5.2, *p* = .004), working or studying (adjusted OR = 2.8; 95%CI = 1.5–5.0, *p* = .001), reading a newspaper daily (adjusted OR = 4.4; 95%CI = 1.7–11.2, *p* = .002) and with several risk behaviors, such as a daily/weekly alcohol consumption (adjusted OR = 3.3; 95%CI = 1.8–6.1, *p* < .001), reporting early sex (adjusted OR = 2.3; 95%CI = 1.1–5.0, *p* = .04), having multiple sexual partners (adjusted OR = 4.1; 95%CI = 2.2–7.7, *p* < .001) and having extragenital sexual practices (adjusted OR = 2.4; 95%CI = 1.3–4.4, *p* = .006) (Table 2 and Fig. 2). Reporting a high religiosity was associated with a lower frequency of paid sex (among men this association was statistically significant: OR = 0.1; 95%CI = 0.02–0.7, *p* = .02). In the crude analysis, belonging to the *Èglise de réveil* compared to Christians was associated with a lower prevalence of paid sex, however, significance was lost in the multivariate analysis. When the association analysis was stratified by sex, an interaction was observed. Stronger associations were found among women for newspaper reading (adjusted OR = 8.2; 95%CI = 1.0–68.4, *p* = .05), alcohol consumption (adjusted OR = 13.5; 95%CI = 3.5–52.4, *p* < .001) and multiple partnerships (adjusted OR = 13.6; 95%CI = 3.6–51.6, *p* < .001) (Table 2). Among women, studying or working was inversely associated with paid sex, although this association was not significant.

**Table 2:** Factors associated with reporting paid sex at baseline and at 6-month follow-up

	Paid sex at baseline (*n* = 73)				Paid sex at 6-month follow-up (*n* = 16)	
	cOR (95%CI)	aOR (95%CI)*	aOR(95%CI) Women (*n* = 17)	aOR(95%CI) Men (*n* = 56)	cOR(95%CI)	aOR(95%CI)**
*Baseline Sociodemographics*						
Sex (men)(vs. women)	**5.1 (2.9–9.0)**	**2.7 (1.4–5.2)**	–	–	**2.9 (1.0–8.5)**	1.4 (0.4–4.9)
Age (adults) (vs. 15-24 years)	**3.1 (1.5–6.3)**	1.6 (0.7–3.8)	1.4 (0.3–5.8)	1.6 (0.5–5.1)	3.0 (0.7–13.3)	1.9 (0.4–10.4)
University studies (vs. secondary)	0.9 (0.6–1.7)	–	–	–	1.1 (0.4–3.3)	–
Working or studying (vs. unemployed)	**2.3 (1.0–5.3)**	**2.8 (1.5–5.0)**	0.8 (0.2–3.1)	**4.1 (1.9–8.4)**	2.1 (0.7–6.1)	2.4 (0.4–14.3)
Daily access to 4–6 mass media	**2.2 (1.2–3.9)**	–	–	–	1.2 (0.3–4.4)	–
Daily reading of newspaper	**2.8 (1.3–6.2)**	**4.4 (1.7–11.2)**	**8.2 (1.0–68.4)**	**3.6 (1.2–10.8)**	NA	NA
Daily/weekly use of the Internet	**2.0 (1.0–4.0)**	–	–	–	3.1 (0.7–13.7)	–
Église de réveil (vs. Christian)	**0.5 (0.3–0.9)**	–	–	–	**0.2 (0.0–1.0)**	0.4 (0.1–1.9)
Religiosity (daily/weekly praying)	**0.1 (0.0–0.2)**	**0.1 (0.0–0.4)**	0.1 (0.0–3.7)	**0.1 (0.0–0.7)**	NA	NA
Married (vs. single)	**1.9 (1.1–3.2)**	0.9 (0.4–1.7)	2.3 (0.5–12.0)	0.9 (0.4–1.8)	1.5 (0.5–4.6)	–
*Baseline Risk Behaviors*						
Alcohol consumption (daily/weekly)	**4.9 (3.0–8.2)**	**3.3 (1.8–6.1)**	**13.5 (3.5–52.4)**	**2.2 (1.1–4.5)**	**6.1 (2.2–17.2)**	**3.5 (1.1–11.0)**
First sexual intercourse before 15 years	**1.9 (1.0–3.6)**	**2.3 (1.1–5.0)**	2.8 (0.5–16.81)	2.3 (0.9–5.8)	2.1 (0.6–7.9)	2.0 (0.5–8.9)
Multiple sexual partners	**2.4 (1.4–4.1)**	–	–	–	1.7 (0.6–5.1)	–
Multiple partners in previous 6 months	**8.2 (4.8–13.9)**	**4.1 (2.2–7.7)**	**13.6 (3.6–51.6)**	**3.2 (1.5–6.9)**	**5.3 (1.9–15.1)**	2.8 (0.8–10.0)
Concurrent partners in previous 6 months	**5.9 (3.4–10.3)**	–	–	–	**7.7 (2.1–27.7)**	–
Condom use (vs. non-use)	**7.4 (1.8–30.6)**	–	–	–	NA	NA
Oral/Anal sex	**3.9 (2.4–6.4)**	**2.4 (1.3–4.4)**	**3.5 (1.0–11.9)**	**2.2 (1.1–4.6)**	**3.4 (1.2–9.3)**	2.0 (0.6–6.3)
HIV misconceptions	1.2 (0.7–2.1)	–	–	–	2.1 (0.8–6.1)	–
HIV test (+ /indeterminate) (vs. negative)	**2.8 (1.4–5.7)**	**3.2 (1.3–7.8**)	**10.7 (2.1–53.8)**	1.9 (0.6–5.6)	1.2 (0.2–9.7)	–
Previously HIV tested	0.9 (0.6–1.7)	–	–	–	0.5 (0.2–1.3)	–
HIV risk perceived	**2.2 (1.3–3.7)**	–	–	–	2.0 (0.7–6.1)	–
Risk behaviors at 6-month follow-up						
Alcohol consumption (daily/weekly)	**NA**				**3.6 (1.3–10.0)**	1.1 (0.3–4.3)
Multiple sexual partners at 6-month follow-up					**9.2 (3.2–26.5)**	
Multiple partners in previous 6 months					**16.1 (5.0–52.2)**	**9.5 (2.3–40.0)**
Concurrent partners in previous 6 months					**13.1 (3.6–47.3)**	
Oral sex in previous 6 months					**15.4 (2.0–118.0)**	**16.0 (1.9–137.0)**
Anal sex in previous 6 months					**3.9 (1.3–11.2)**	
Perceived HIV risk at 6-month follow-up					**5.0 (1.7–14.5)**	3.4 (0.9–13.0)

cOR: crude Odds Ratio; aOR: adjusted OR; N/A: not applicable

Bold values indicate statistically significant

*Multivariate logistic regression adjusted for sex, age, working/studying, daily newspaper reading, daily/weekly church attendance/praying, marital status (married), daily/weekly alcohol consumption, first sex before 15 years, multiple sexual partners, extragenital sexual practices and HIV test result

**Sex-stratified analysis could not be carried out at 6-month follow-up due to the small sample size

When the association between baseline variables and paid sex at 6-month follow-up was analyzed, the same factors remained significantly associated in the crude analyses. However, as a result of the low participants' sample size reporting paid sex at 6-month follow-up (*n* = 16), only alcohol consumption remained significant in the adjusted model (adjusted OR = 3.5; 95%CI = 1.1–11.0, *p* = .034) (Table 2).

In the 6-month follow-up questionnaire information on risk behaviors in the previous 6 months was collected, and alcohol consumption (OR = 3.6; 95%CI = 1.3–10.1, *p* = .013) and extragenital sex remained significantly associated with paid sex at 6-month follow-up (OR = 16.0; 95%CI = 1.9–137.0, *p* = .011) (Table 2).

The association with paid sex at 12 months was not analyzed as only 3 participants were reporting paid sex at that follow-up.

### HIV Diagnosis and Reported STIs

Three percent of the study population got a new positive HIV diagnosis at baseline and 10% of the participants reported having been diagnosed with an STI in the last year. Overall, only 3% perceived a high HIV risk (Table 1).

### Association Between Paid Sex and HIV

When the association between paid sex and a positive/undetermined HIV test at baseline was analyzed, a non-significant association was found for men (aOR = 1.9; 95%CI: 0.6–5.6, *p* = .286) and a strong and significant association was present for women (aOR = 10.7; 95%CI: 2.1–53.8, *p* = .005) (Table 2).

## Discussion

### Prevalence of Paid Sex

Between 2016 and 2018, a high prevalence of paid sex was reported among men and women from the general population attending HIV VCT in Kinshasa. Paid sex was associated with other consumption and sexual risk behaviors.

Having ever had paid sex was reported by 18% of men and 4% of women. The prevalence of paid sex was lower at follow-up (there was a higher attrition of people engaged in paid sex).

Apart from the official data from the National Demographic and Health Survey (DHS) (Hodgins et al., 2022), few studies have evaluated the prevalence of paid sex among people from the general population in SSA (Baltazar et al., 2021; Kloek et al., 2022; Oldenburg et al., 2014). For the DRC the only previous data in the country on this exchanged sex was collected at the DHS in 2013–14 (DHS, 2014). The survey showed that 23% of men aged 15–49 years from Kinshasa reported having “ever” had paid sex and 8% when it was referred to the previous year. This prevalence is slightly higher than our 18% prevalence for men. This could be a result of the different areas in Kinshasa participating in the DHS, far more numerous and diverse than those included in OKAPI. Considering the different ages of the participants (15–49 years in the DHS and 15–69 years in OKAPI) we estimate this did not have an impact, as only a few men over 49 years reported paid sex in our study.

Based on other DHS results from different SSA countries, the mean prevalence of paid sex among men was 10%; the DRC was, after Madagascar, the country with the second highest frequency of paid sex among men. No data are available in the DHS for women. A study carried out among women in South Africa found that 21% reported having ever had sex with a non-primary male partner in exchange for material goods or money (Dunkle et al., 2004). In Zimbabwe, Ruangtragool et al. (2022) analyzed the prevalence of paid sex among men and women from the general population and found that 6% of men and 3% of women reported transactional sex in the last 12 months.

Currently, VCT at Monkole Hospital and most centers within the National AIDS Program do not include paid sex in their counseling sessions. The high prevalence of paid sex found in this study highlights the need to include this specific aspect in the counseling messages, to improve the routine prevention strategies in Kinshasa.

### Factors Associated with Paid Sex

Consistently with our results, other studies in SSA have shown a higher frequency of paid sex among men (Adjei, 2017; Krisch et al., 2019; Wamoyi et al., 2019). Among women not self-identified as sex workers, paid sex is usually related to material needs, such as food, clothing, transport, items for their children or families, or even somewhere to sleep (Dunkle et al., 2004; Lusey et al., 2014). For men, as shown in different parts of the world and not just in African countries, transactional sex is often linked to an erroneous interpretation of masculinity (Deogan et al., 2021; Huysamen et al., 2015; Shumka et al., 2017). In the African culture where men’s dominance over women is more present than in other cultures, it is even easier to find this behavior (Conroy et al., 2016; Duby et al., 2023; UNAIDS, 2022b). Thus, working with men on the particular reasons for having paid sex is still necessary and VCT sessions can be an opportunity for this health educational approach.

Being working or studying and reading a newspaper daily were independently associated with reporting paid sex. Both variables are related to a higher economic status. As shown in other studies, access to paid sex is more frequent among those people who can afford this extraordinary payment (Chikutsa et al., 2015; DHS 2014; Dunkle et al., 2004; Jewkes et al., 2011; Krisch et al., 2019; Lusey et al., 2014; Mbonye et al., 2022; Seidu et al., 2019). On the other hand, among women, working or studying has an inverse association with paid sex which can be probably explained by the fact that having a stable status prevents women from having sex for money and material goods they need.

In the crude analyses, access to other mass media different from the newspaper or daily/weekly use of the Internet were associated with a higher frequency of paid sex, but significance was lost in the adjusted analyses. These media can be a way in which paid sex can be promoted. However, public health preventive strategies, can be a good option for promoting behavioral changes. Thus, considering that almost all participants reported daily access to their mobile phones, strategies based on phone use could help reduce the prevalence of this risk behavior.

Reporting daily/weekly praying or religious service attendance was independently associated with a lower frequency of paid sex. Participants who belonged to the *Église de réveil* reported less paid sex than participants from other religions. This could be a result of under-reporting as they could feel they could not report something stigmatizing. However, people belonging to this religious group usually have a lower economic level and this finding is consistent with the previously shown fact that a higher economic level is associated with higher access to paid sex. On the other hand, a high religiosity remained inversely associated with reporting paid sex, after adjusting for economic status. As described for other healthy behaviors, the effects of religious practice can be a result of their positive emotional or social impact rather than a consequence of normative aspects specific to certain religions (Ahrenfeldt et al., 2023).

In the present study, alcohol consumption was associated with reporting paid sex, both at baseline and follow-up, and this association was much stronger for women. This is consistent with other studies from SSA countries (Krisch et al., 2019; Tran et al., 2019; Tumwesigye et al., 2012*).* The DRC is one of the African countries with the lowest gross domestic product and with lower alcohol consumption (Ritchie & Roser, 2018; WHO, 2018). However, similar to the official estimates, in our study population 24 and 12% of men and women, respectively, reported daily or weekly alcohol consumption. It has been widely explained that alcohol consumption is associated with sexual risk behaviors. A recent publication has shown that it is not just the type of drink or the drinking amount that increases the odds of sexual activity but also where you drink. For example, parties or bars are considered environments with an increased risk (Hone et al., 2023). Alcohol prevention strategies to reduce the associated risks in the Congolese population should consider the entire context of alcohol consumption.

As described in a previous OKAPI analysis (Carlos et al., 2021), there was a very low retention rate at the 12-month follow-up. Among other factors, alcohol consumption was inversely associated with retention. As people reporting paid sex were more likely to report alcohol consumption, this could explain part of the higher attrition among people reporting paid sex.

Another risk behavior associated with paid sex, both in the cross-sectional and the longitudinal analyses, was reporting multiple sexual partners, as shown in other studies from Africa (Baltazar et al., 2021; Krisch et al., 2019; Ssempijja, 2022). This effect was stronger for women. Both sexual risks are connected. As previously reported in this cohort, different sexual risk behaviors are associated with each other (Carlos S, et al., 2021). In the present study other sexual behaviors, such as reporting extragenital practices (oral or anal sex) or inconsistent condom use were associated with paid sex. The overlapping of different risk behaviors among the population, which increases the risk of acquiring or transmitting HIV and other STI, should all be considered in the counseling sessions (Waters & Dewsnap, 2022).

### Paid Sex and HIV

As expected and described in the literature (Baltazar et al., 2021), participants reporting paid sex were more likely to have an HIV-positive test. This fact should lead the Congolese government and HIV organizations to support preventive campaigns that promote the avoidance of paid sex. Raising awareness of its risks in healthcare settings is necessary to reduce the incidence of HIV and other STIs.

### Study Limitations

The present study has some limitations. First, paid sex and other risk behaviors are socially undesirable, and thus, misclassification bias could be present. Although professional interviewers (male to male, female to female), individual rooms, and anonymity were available at baseline and both follow-ups, to reduce this bias, we acknowledge that it is difficult to mitigate. If this bias was present, it would be non-differential and results would have been biased toward the null. Furthermore, the counseling received at baseline about the negative effects of sexual risk behaviors could influence the biased responses on sexual behaviors but also, the other way round, on reducing these risk behaviors. Secondly, the question used about exposure to paid sex was quite general and did not collect specific information about what they considered paid sex (ie. people reporting paid sex may have not had risky sex but other kinds of paid sex more related to pornography), the type of partners involved, or even the reasons for having paid sex. In this sense, the analyses adequately answer the study objectives (to evaluate the overall prevalence and associated factors). However, additional aspects need to be considered. Adding qualitative analyses in the future can also help to complete this information. Third, it needs to be highlighted the potential for bias in the fact that people reporting paid sex were less likely to be retained. Due to the result of the low retention rate at 12-month follow-up, no longitudinal analyses could be carried out to evaluate the association between paid sex at 12-month follow-up and participants’ characteristics. However, they could be done for the 6-month follow-up and the same risk behaviors as at baseline showed to be associated with paid sex.

### Study Strengths

Despite the mentioned limitations, this study has several strengths. First, this is the first study evaluating the prevalence of paid sex among people from the general population attending HIV VCT in Kinshasa, and the first time that data on women were collected. Second, both cross-sectional and longitudinal analyses were carried out to evaluate the different associations and all of them clearly showed the strong association between paid sex and male sex and with other risk behaviors such as alcohol consumption, multiple sexual partnerships, and extragenital sexual practices. Finally, the study included nearly 800 participants at baseline which allowed multivariate logistic regressions to be carried out, taking into account the possible effect of many sociodemographic, knowledge, and behavioral variables.

### Conclusion

In conclusion, paid sex is a prevalent sexual practice among Congolese men and women from the general population and it is associated with alcohol consumption and other sexual behaviors. It needs to be included among the risk factors to be mentioned in the HIV counseling sessions in the Democratic Republic of the Congo.

## Supplementary Information

Below is the link to the electronic supplementary material.

Supplementary file1 (DOCX 22 KB)

## Data Availability

The underlying data set necessary for the replication of this study, as well as the main analyses syntaxis and the codebook of variables and labels, are available within Harvard Dataverse. The original questionnaires are available as supplementary material.

## References

[CR2] Adal, M. (2019). Systematic review on HIV situation in Addis Ababa. *Ethiopia BMC Public Health,**19*(1), 1544. 10.1186/s12889-019-7885-8 31752778 10.1186/s12889-019-7885-8 PMC6873765

[CR3] Adjei, J. K., & Saewyc, E. M. (2017). Boys are not exempt: Sexual exploitation of adolescents in sub-Saharan Africa. *Child Abuse & Neglect,**65*, 14–23. 10.1016/j.chiabu.2017.01.001 28110108 10.1016/j.chiabu.2017.01.001

[CR4] Ahinkorah, B. O., Budu, E., Seidu, A. A., Hagan, J. E., Jr., Agbaglo, E., Hormenu, T., Schack, T., & Yaya, S. (2020). Consistent condom use among men who pay for sex in sub-Saharan Africa: empirical evidence from demographic and health surveys. *PLoS ONE,**15*(8), e0236552. 10.1371/journal.pone.0236552 32776965 10.1371/journal.pone.0236552 PMC7416936

[CR5] Ahrenfeldt, L. J., Möller, S., Hvidt, N. C., VanderWeele, T. J., & Stripp, T. A. (2023). Effect of religious service attendance on mortality and hospitalisations among Danish men and women: Longitudinal findings from REGLINK-SHAREDK. *European Journal of Epidemiology,**38*(3), 281–289. 10.1007/s10654-023-00964-y 36646924 10.1007/s10654-023-00964-y

[CR6] Baltazar, C. S., Mehta, N., Juga, A., Boothe, M., Langa, D. C., Simbine, P., & Kellogg, T. A. (2021). Who are the men who pay for sex in Mozambique? Results from the National HIV/AIDS Indicator Survey 2015. *Archives of Sexual Behavior,**50*(5), 2057–2065. 10.1007/s10508-020-01892-8 33821377 10.1007/s10508-020-01892-8 PMC11923418

[CR8] Bruni, L., Albero, G., Serrano, B., Mena, M., Collado, J. J., Gómez, D., Muñoz, J., Bosch, F. X., de Sanjosé, S. (2021). *ICO/IARC Information Centre on HPV and Cancer (HPV Information Centre)*. *Human Papillomavirus and Related Diseases in the World*. Summary Report 22

[CR9] Carlos, S., Martínez-González, M. A., Burgueño, E., López-Del Burgo, C., Ruiz-Canela, M., Ndarabu, A., Tshilolo, L., Tshiswaka, P., Labarga, P., & de Irala, J. (2015). Misconceptions about HIV infection in Kinshasa (Democratic Republic of Congo): A case-control study on knowledge, attitudes and practices. *Sexually Transmitted Infections,**91*(5), 334–337. 10.1136/sextrans-2014-051734 25416838 10.1136/sextrans-2014-051734

[CR10] Carlos, S., Nzakimuena, F., Reina, G., Lopez-Del Burgo, C., Burgueño, E., Ndarabu, A., Osorio, A., & de Irala, J. (2016). Factors that lead to changes in sexual behaviours after a negative HIV test: Protocol for a prospective cohort study in Kinshasa. *BMC Public Health,**16*, 606. 10.1186/s12889-016-3285-5 27439981 10.1186/s12889-016-3285-5 PMC4955130

[CR11] Carlos, S., Lopez-Del Burgo, C., Burgueño, E., Martinez-Gonzalez, M. A., Osorio, A., Ndarabu, A., Passabosc, C., & de Irala, J. (2017). Male condom use, multiple sexual partners and HIV: A prospective case-control study in Kinshasa (DRC). *AIDS Care,**29*(6), 772–781. 10.1080/09540121.2016.1258450 27852108 10.1080/09540121.2016.1258450

[CR12] Carlos, S., López-Del Burgo, C., Ndarabu, A., Osorio, A., Rico-Campà, A., Reina, G., Burgueño, E., & de Irala, J. (2019). Heterosexual oral and anal sex in Kinshasa (D. R. Congo): Data from OKAPI prospective cohort. *PLoS ONE,**14*(1), e0210398. 10.1371/journal.pone.0210398 30650137 10.1371/journal.pone.0210398 PMC6334946

[CR13] Carlos, S., Burgueño, E., Ndarabu, A., Reina, G., Lopez-Del Burgo, C., Osorio, A., Makonda, B., & de Irala, J. (2021). Predictors of retention in the prospective HIV prevention OKAPI cohort in Kinshasa. *Scientific Reports,**11*(1), 5431. 10.1038/s41598-021-84839-w 33686218 10.1038/s41598-021-84839-w PMC7970874

[CR14] Chatsika, Z. J., Kumitawa, A., Samuel, V., Azizi, S. C., & Jumbe, V. C. (2020). Voluntary medical male circumcision and sexual practices among sexually active circumcised men in Mzuzu, Malawi: A cross-sectional study. *BMC Public Health,**20*(1), 211. 10.1186/s12889-020-8309-5 32046686 10.1186/s12889-020-8309-5 PMC7014635

[CR15] Chikutsa, A., Ncube, A. C., & Mutsau, S. (2015). Association between wanting circumcision and risky sexual behaviour in Zimbabwe: Evidence from the 2010–11 Zimbabwe Demographic and Health Survey. *Reproductive Health,**12*, 15. 10.1186/s12978-015-0001-3 25889318 10.1186/s12978-015-0001-3 PMC4364469

[CR16] Conroy, A. A., Tsai, A. C., Clark, G. M., Boum, Y., Hatcher, A. M., Kawuma, A., Hunt, P. W., Martin, J. N., Bangsberg, D. R., & Weiser, S. D. (2016). Relationship power and sexual violence among HIV-positive women in rural Uganda. *AIDS and Behavior,**20*(9), 2045–2053. 10.1007/s10461-016-1385-y 27052844 10.1007/s10461-016-1385-y PMC4996683

[CR17] Deogan, C., Jacobsson, E., Mannheimer, L., & Björkenstam, C. (2021). Are men who buy sex different from men who do not?: Exploring sex life characteristics based on a randomized population survey in Sweden. *Archives of Sexual Behavior,**50*(5), 2049–2055. 10.1007/s10508-020-01843-3 33354757 10.1007/s10508-020-01843-3 PMC8275502

[CR18] Döring, N., Walter, R., Mercer, C. H., Wiessner, C., Matthiesen, S., & Briken, P. (2022). Men who pay for sex: Prevalence and sexual health. *Deutsches Arzteblatt International,**119*(12), 201–207. 10.3238/arztebl.m2022.0107 35019837 10.3238/arztebl.m2022.0107 PMC9277131

[CR19] Duby, Z., Bergh, K., Jonas, K., Reddy, T., Bunce, B., Fowler, C., & Mathews, C. (2023). Men rule this is the normal thing. We normalise it and it’s wrong: Gendered power in decision-making around sex and condom use in heterosexual relationships amongst Adolescents and Young People in South Africa. *AIDS and Behavior,**27*(6), 2015–2029. 10.1007/s10461-022-03935-8 36441410 10.1007/s10461-022-03935-8 PMC10149448

[CR20] Dunkle, K. L., Jewkes, R. K., Brown, H. C., Gray, G. E., McIntryre, J. A., & Harlow, S. D. (2004). Transactional sex among women in Soweto, South Africa: Prevalence, risk factors and association with HIV infection. *Social Science and Medicine,**59*(8), 1581–1592. 10.1016/j.socscimed.2004.02.003 15279917 10.1016/j.socscimed.2004.02.003

[CR21] Foley, E. E., & Drame, F. M. (2013). Mbaraan and the shifting political economy of sex in urban Senegal. *Culture, Health & Sexuality,**15*(2), 121–134. 10.1080/13691058.2012.744849 10.1080/13691058.2012.744849 23181265

[CR22] Green, E. C., & Ruark, A. H. (2011). *AIDS, behavior and culture: Understanding evidence-based prevention*. Left Coast Press.

[CR23] Hodgins, C., Stannah, J., Kuchukhidze, S., Zembe, L., Eaton, J. W., Boily, M. C., & Maheu-Giroux, M. C. (2022). Population sizes, HIV prevalence, and HIV prevention among men who paid for sex in sub-Saharan Africa (2000–2020): A meta-analysis of 87 population-based surveys. *PLoS Medicine,**19*(1), e1003861. 10.1371/journal.pmed.1003861 35077459 10.1371/journal.pmed.1003861 PMC8789156

[CR24] Hone, L. S. E., Testa, M., & Wang, W. (2023). It’s not just drinking, but where you drink: A daily diary study of drinking venue effects on sexual activity with new partners. *Addictive Behaviors,**140*, 107607. 10.1016/j.addbeh.2023.107607 36652812 10.1016/j.addbeh.2023.107607

[CR25] Hosmer, D. W., Lemeshow, S., & Sturdivant, R. X. (2013). *Applied logistic regression*. Wiley.

[CR26] Huysamen, M., & Boonzaier, F. (2015). Men’s constructions of masculinity and male sexuality through talk of buying sex. *Culture, Health & Sexuality,**17*(5), 541–554. 10.1080/13691058.2014.963679 10.1080/13691058.2014.963679 25287270

[CR27] James, C., Harfouche, M., Welton, N. J., Turner, K. M., Abu-Raddad, L. J., Gottlieb, S. L., & Looker, K. J. (2020). Herpes simplex virus: Global infection prevalence and incidence estimates, 2016. *Bulletin of the World Health Organization,**98*(5), 315–329. 10.2471/BLT.19.237149 32514197 10.2471/BLT.19.237149 PMC7265941

[CR28] Jewkes, R., Sikweyiya, Y., Morrell, R., & Dunkle, K. (2011). Gender inequitable masculinity and sexual entitlement in rape perpetration South Africa: Findings of a cross-sectional study. *PLoS ONE,**6*(12), e29590. 10.1371/journal.pone.0029590 22216324 10.1371/journal.pone.0029590 PMC3247272

[CR29] Kayembe, P. K., Mapatano, M. A., Busangu, A. F., Nyandwe, J. K., Musema, G. M., Kibungu, J. P., Mashinda, D. K., Matamba, L. T., & Mayala, G. M. (2008). Determinants of consistent condom use among female commercial sex workers in the Democratic Republic of Congo: Implications for interventions. *Sexually Transmitted Infections, 84*(3), 202–206. 10.1136/sti.2007.028324 10.1136/sti.2007.028324 18055581

[CR30] Kiyingi, J., Nabunya, P., Bahar, O. S., Mayo-Wilson, L. J., Tozan, Y., Nabayinda, J., Namuwonge, F., Nsubuga, E., Kizito, S., Nattabi, J., Nakabuye, F., Kagayi, J., Mwebembezi, A., Witte, S. S., & Ssewamala, F. M. (2022). Prevalence and predictors of HIV and sexually transmitted infections among vulnerable women engaged in sex work: Findings from the Kyaterekera Project in Southern Uganda. *PLoS ONE,**17*(9), e0273238. 10.1371/journal.pone.0273238 36174054 10.1371/journal.pone.0273238 PMC9522279

[CR31] Kloek, M., Bulstra, C. A., Chabata, S. T., Fearon, E., Taramusi, I., de Vlas, S. J., Cowan, F. M., & Hontelez, J. A. C. (2022). No increased HIV risk in general population near sex work sites: A nationally representative cross-sectional study in Zimbabwe. *Tropical Medicine & Interantional Health,**27*(8), 696–704. 10.1111/tmi.13791 10.1111/tmi.13791 PMC9545096 35687493

[CR32] Krisch, M., Averdijk, M., Valdebenito, S., & Eisner, M. (2019). Sex trade among youth: A global review of the prevalence, contexts and correlates of transactional sex among the general population of youth. *Adolescent Research Review,**4*, 115–134. 10.1007/s40894-019-00107-z 10.1007/s40894-019-00107-z

[CR33] Kyegombe, N., Stoebenau, K., Chimbindi, N., Zuma, T., Shahmanesh, M., Seeley, J., & Wamoyi, J. (2021). Measuring transactional sex in different contexts: How do tools to measure this practice perform in rural South Africa? *African Journal of AIDS Research,**20*(4), 329–335. 10.2989/16085906.2021.2012213 34905457 10.2989/16085906.2021.2012213

[CR34] Longo, J. D., Simaléko, M. M., Ngbale, R., Grésenguet, G., Brücker, G., & Bélec, L. (2017). Spectrum of female commercial sex work in Bangui Central African Republic. *SAHARA-J: Journal of Social Aspects of HIV/AIDS,**14*(1), 171–184. 10.1080/17290376.2017.1394907 10.1080/17290376.2017.1394907 PMC5678296 29092678

[CR35] Looker, K. J., Welton, N. J., Sabin, K. M., Dalal, S., Vickerman, P., Turner, K. M. E., Boily, M. C., & Gottlieb, S. L. (2020). Global and regional estimates of the contribution of herpes simplex virus type 2 infection to HIV incidence: A population attributable fraction analysis using published epidemiological data. *Lancet Infectious Diseases,**20*(2), 240–249. 10.1016/S1473-3099(19)30470-0 31753763 10.1016/S1473-3099(19)30470-0 PMC6990396

[CR36] Lusey, H., San Sebastian, M., Christianson, M., Dahlgren, L., & Edin, K. E. (2014). Conflicting discourses of church youths on masculinity and sexuality in the context of HIV in Kinshasa Democratic Republic of Congo. *SAHARA-J: Journal of Social Aspects of HIV/AIDS,**11*(1), 84–93. 10.1080/17290376.2014.930695 10.1080/17290376.2014.930695 PMC4272190 25000272

[CR37] Maher, A. D., Nakanyala, T., Mutenda, N., Banda, K. M., Prybylski, D., Wolkon, A., Jonas, A., Sawadogo, S., Ntema, C., Chipadze, M. R., Sinvula, G., Tizora, A., Mwandemele, A., Chaturvedi, S., Agovi, A. M. A., Agolory, S., Hamunime, N., Lowrance, D. W., Mcfarland, W., & Patel, S. V. (2020). Rates and correlates of HIV incidence in Namibia’s Zambezi region from 2014 to 2016: Sentinel, Community-Based Cohort Study. *JMIR Public Health and Surveillance,**6*(2), e17107. 10.2196/17107 32348290 10.2196/17107 PMC7381049

[CR38] Mantell, J., Franks, J., Zerbe, A., Lamb, M. R., Reed, D. M., Omollo, D., Lahuerta, M., Naitore, D., El-Sadr, W. M., & Agot, K. (2022). MPrEP+ study protocol: A prospective cohort study assessing the feasibility and acceptability of an HIV pre-exposure prophylaxis (PrEP) strategy for male clients of female sex workers in Kisumu, Kenya. *BMJ Open,**12*(11), e064037. 10.1136/bmjopen-2022-064037 36332953 10.1136/bmjopen-2022-064037 PMC9639093

[CR39] Mayanja, Y., Kamacooko, O., Lunkuse, J. F., Kyegombe, N., & Ruzagira, E. (2023). Prevalence, perpetrators, and factors associated with intimate partner violence among adolescents living in urban slums of Kampala, Uganda. *Journal of Interpersonal Violence,**38*(13–14), 8377–8399. 10.1177/08862605231155128 36825721 10.1177/08862605231155128

[CR40] Mbonye, M., Siu, G., & Seeley, J. (2022). The meaning of fatherhood to men in relationships with female sex workers in Kampala, Uganda: The struggle to model the traditional parameters of fatherhood and masculinity. *PLoS ONE,**17*(8), e0273298. 10.1371/journal.pone.0273298 36044520 10.1371/journal.pone.0273298 PMC9432681

[CR41] McMillan, K., Worth, H., & Rawstorne, P. (2018). Usage of the terms prostitution sex work, transactional sex, and survival sex: Their utility in HIV prevention research. *Archives of Sexual Behavior,**47*(5), 1517–1527. 10.1007/s10508-017-1140-0 29305773 10.1007/s10508-017-1140-0

[CR42] Mihretie, G. N., Kassa, B. G., Ayele, A. D., Liyeh, T. M., Belay, H. G., Miskr, A. D., Minuye, B., Azanaw, M. M., & Worke, M. D. (2023). Transactional sex among women in Sub-Saharan Africa: A systematic review and meta-analysis. *PLoS ONE,**18*(6), e0286850. 37289839 10.1371/journal.pone.0286850 PMC10249834

[CR43] Ntumbanzondo, M., Dubrow, R., Niccolai, L. M., Mwandagalirwa, K., & Merson, M. H. (2006). Unprotected intercourse for extra money among commercial sex workers in Kinshasa, Democratic Republic of Congo. *AIDS Care,**18*(7), 777–785. 10.1080/09540120500412824 16971288 10.1080/09540120500412824

[CR44] Oldenburg, C. E., Perez-Brumer, A. G., Reisner, S. L., & Mattie, J. (2014). Global burden of HIV among men who engage in transactional sex: A systematic review and meta-analysis. *PLoS ONE,**9*(7), e103549. 10.1371/journal.pone.0103549 25068720 10.1371/journal.pone.0103549 PMC4113434

[CR45] Ortblad, K. F., Musoke, D. K., Ngabirano, T., Salomon, J. A., Haberer, J. E., McConnell, M., Oldenburg, C. E., & Bärnighausen, T. (2019). Is knowledge of HIV status associated with sexual behaviours? A fixed effects analysis of a female sex worker cohort in urban Uganda. *Journal of International AIDS Society,**22*(7), e25336. 10.1002/jia2.25336 10.1002/jia2.25336 PMC6615530 31287625

[CR46] Ministère du Plan et Suivi de la Mise en oeuvre de la Révolution de la Modernité (MPSMRM), Ministère de laSanté Publique (MSP) et ICF International. (2014). Enquête Démographique et de Santé en République Démocratique du Congo 2013–2014. Rockville, MD: MPSMRM, MSP et ICF International.

[CR1] Ritchie, H., & Roser, M. (2018). "Alcohol Consumption". *OurWorldInData.org*. Retrieved from: 'https://ourworldindata.org/alcohol-consumption'.

[CR47] Ruangtragool, L., Silver, R., Machiha, A., Gwanzura, L., Hakim, A., Lupoli, K., Musuka, G., Patel, H., Mugurungi, O., Tippett Barr, B. A., & Rogers, J. H. (2022). Factors associated with active syphilis among men and women aged 15 years and older in the Zimbabwe population-based HIV impact assessment (2015–2016). *PLoS ONE,**17*(3), e0261057. 10.1371/journal.pone.0261057 35298475 10.1371/journal.pone.0261057 PMC8929562

[CR48] Sangwa-Lugoma, G., Ramanakumar, A. V., Mahmud, S., Liaras, J., Kayembe, P. K., Tozin, R. R., Lorincz, A., & Franco, E. L. (2011). Prevalence and determinants of high-risk human papillomavirus infection in women from a sub-Saharan African community. *Sexually Transmitted Diseases,**38*(4), 308–315. 10.1097/OLQ.0b013e3181fc6ec0 21150817 10.1097/OLQ.0b013e3181fc6ec0

[CR49] Seidu, A. A., Darteh, E. K., Kumi-Kyereme, A., Dickson, K. S., & Ahinkorah, B. O. (2020). Paid sex among men in sub-Saharan Africa: Analysis of the Demographic and Health Survey. *SSM-Population Health,**11*, 100459. 10.1016/j.ssmph.2019.100459 32875050 10.1016/j.ssmph.2019.100459 PMC7451820

[CR50] Shumka, L., Strega, S., & Hallgrimsdottir, H. K. (2017). “I wanted to feel like a man again”: Hegemonic masculinity in relation to the purchase of street-level sex. *Frontiers in Sociology,**2*, 15. 10.3389/fsoc.2017.00015 10.3389/fsoc.2017.00015

[CR51] Silva, S., Ayoub, H. H., Johnston, C., Atun, R., & Abu-Raddad, L. J. (2022). Estimated economic burden of genital herpes and HIV attributable to herpes simplex virus type 2 infections in 90 low- and middle-income countries: A modeling study. *PLoS Medicine,**19*(12), e1003938. 10.1371/journal.pmed.1003938 36520853 10.1371/journal.pmed.1003938 PMC9754187

[CR52] Ssempijja, V., Nakigozi, G., Ssekubugu, R., Kagaayi, J., Kigozi, G., Nalugoda, F., Nantume, B., Batte, J., Kigozi, G., Yeh, P. T., Nakawooya, H., Serwadda, D., Quinn, T. C., Gray, R. H., Wawer, M. J., Grabowski, K. M., Chang, L. W., Van’t Hoog, A., Cobelens, F., & Reynolds, S. J. (2022). High rates of pre-exposure prophylaxis eligibility and associated HIV incidence in a population with a generalized HIV epidemic in Rakai, Uganda. *Journal of Acquired Immune Deficiency Syndrome,**90*(3), 291–299. 10.1097/QAI.0000000000002946 10.1097/QAI.0000000000002946 PMC9177156 35259129

[CR53] Steen, R., Wheeler, T., Gorgens, M., Mziray, E., & Dallabetta, G. (2015). Feasible, efficient and necessary, without exception—working with sex workers interrupts HIV/STI transmission and brings treatment to many in need. *PLoS ONE,**10*(10), e0121145. 10.1371/journal.pone.0121145 26488796 10.1371/journal.pone.0121145 PMC4619404

[CR54] Stoebenau, K., Dunkle, K., Willan, S., Shai, N., & Gibbs, A. (2023). Assessing risk factors and health impacts across different forms of exchange sex among young women in informal settlements in South Africa: A cross-sectional study. *Social Science & Medicine,**318*, 115637. 10.1016/j.socscimed.2022.115637 36628880 10.1016/j.socscimed.2022.115637

[CR55] Tran, B. R., Davis, A., Sloan, M., Macera, C., Mbuyi, A. M., & Kabanda, G. K. (2019). Alcohol use and sexual risk behaviors in the Armed Forces of the Democratic Republic of the Congo. *BMC Public Health,**19*(1), 1394. 10.1186/s12889-019-7794-x 31660935 10.1186/s12889-019-7794-x PMC6819617

[CR56] Tumwesigye, N. M., Atuyambe, L., Wanyenze, R. K., Kibira, S. P., Li, Q., Wabwire-Mangen, F., & Wagner, G. (2012). Alcohol consumption and risky sexual behaviour in the fishing communities: Evidence from two fish landing sites on Lake Victoria in Uganda. *BMC Public Health,**11*(12), 1069. 10.1186/1471-2458-12-1069 10.1186/1471-2458-12-1069 PMC3534008 23231779

[CR57] UNAIDS. (2018). *Transactional sex and HIV risk: From analysis to action*. Geneva: Joint United Nations Programme on HIV/AIDS and STRIVE.

[CR59] UNAIDS DATA 2022. (2022a). Geneva: Joint United Nations Programme on HIV/AIDS. 12349391

[CR58] UNAIDS. (2022b). *Dangerous inequalities: World AIDS Day Report 2022*. Geneva: Joint United Nations Programme on HIV/AIDS.

[CR60] Wamoyi, J., Ranganathan, M., Kyegombe, N., & Stoebenau, K. (2019). Improving the measurement of transactional sex in Sub-Saharan Africa: A critical review. *Journal of Acquired Immune Deficiency Syndromes,**80*(4), 367–374. 10.1097/QAI.0000000000001928 30789453 10.1097/QAI.0000000000001928 PMC6410965

[CR61] Waters, L., & Dewsnap, C. (2022). Young women and anal sex: Healthcare professionals must normalise questions about what is normal for many people. *British Medical Journal,**378*, o2323. 10.1136/bmj.o2323 36180093 10.1136/bmj.o2323

[CR62] WHO. (2018). *Global status report on alcohol and health 2018*. Geneva: World Health Organization.

[CR63] WHO. (2021). *Global progress report on HIV, viral hepatitis and sexually transmitted infections, 2021. Accountability for the global health sector strategies 2016–2021: Actions for impact*. Geneva: World Health Organization.

[CR64] Willis, B. (2013). The global public health burden of sex work: A call for research. *The Lancet Global Health,**1*(2), e68. 10.1016/S2214-109X(13)70011-1 25104153 10.1016/S2214-109X(13)70011-1

[CR65] Yang, F., Ketende, S., Mayo-Wilson, L. J., Lyons, C. E., Liestman, B., Diouf, D., Drame, F. M., Coly, K., Turpin, G., Mboup, S., Toure-Kane, C., Castor, D., Cheng, A., Diop-Ndiaye, H., Leye-Diouf, N., Kennedy, C., & Baral, S. (2020). Associations between economic factors and condom use behavior among female sex workers in Dakar and Mbour Senegal. *AIDS and Behavior,**24*(10), 2829–2841. 10.1007/s10461-020-02832-2 32180091 10.1007/s10461-020-02832-2

[CR66] Zhang, J., Ma, B., Han, X., Ding, S., & Li, Y. (2022). Global, regional, and national burdens of HIV and other sexually transmitted infections in adolescents and young adults aged 10–24 years from 1990 to 2019: A trend analysis based on the Global Burden of Disease Study. *The Lancet Child & Adolescent Health,**6*(11), 763–776. 10.1016/S2352-4642(22)00219-X 36108664 10.1016/S2352-4642(22)00219-X

