# Supplementary material for: Prevalence of Paid Sex and Associated Factors Among Women and Men Attending HIV Voluntary Counseling and Testing in Kinshasa, Democratic Republic of the Congo: A Prospective Cohort
Source: Arch Sex Behav. 2024 Aug 15;53(9):3625–37. doi: 10.1007/s10508-024-02939-w (PMC11390826; doi:10.1007/s10508-024-02939-w)
Supplement: Supplementary file 1 — Supplementary file1 (DOCX 22 KB) [file 10508_2024_2939_MOESM1_ESM.docx]

**Supplementary Table. Quality of collected data (missing data and “don´t know or don´t want to answer”).**

|  | **Baseline**  **(N=797)** | | **6-month follow-up**  **(N=456)** | | **12-month follow-up**  **(N=219)** | |
| --- | --- | --- | --- | --- | --- | --- |
|  | Missings  n (%) | Don´t know/  don´t want  n (%) | Missings  n (%) | Don´t know/  don´t want  n (%) | Missings  n (%) | Don´t know/  don´t want  n (%) |
| **Sociodemographics** |  |  |  |  |  |  |
| Sex | - | - | - | - | - | - |
| Age | - | - | - | - | - | - |
| Economic level | - | 11 (1.4) | - | - | - | - |
| Education | - | - | - | - | 3 (1.4) | - |
| Professional status | 373 (47)* | - | - | - | - | - |
| Media access | - | - | - | - | - | - |
| Religion | 373 (47)* | - | - | - | - | - |
| Religiosity (church attendance/praying) | - | - | 151 (33) | - | - | - |
| Married | 1 (0.1) | - | - | - | - | - |
| Living with partner | - | - | - | - | - | - |
| **HIV misconceptions** | - | - | 3 (0.7) | - | - | 8 (3.7) |
| **Risk Behaviors** |  |  |  |  |  |  |
| Alcohol consumption | 59 (7) | - | - | - | - | - |
| Alcohol consumption (binge drinking) | - | - | - | - | - | - |
| Age at first sex | 1 (0.1) | - | - | - | - | - |
| Number of current partners | - | 1 (0.1) | - | - | 8 (5.2) | - |
| Number of partners in last 6 months | - | 1 (0.1) | 1 (0.1) | - | - | - |
| Number of concurrent partners in last 6 m | - | 2 (0.3) | - | - | - | - |
| Condom use | - | - | 2 (0.6) | - | - | - |
| Extragenital practices | - | - | - | 2 (0.6) | - | - |
| Sexual violence | - | - | - | - | - | - |
| **HIV test** |  |  |  |  |  |  |
| **Previous HIV test** | 1 (0.1) | - | - | - | - | - |
| **Some HIV risk perceived** | - | - | - | - | - | - |
| **STI diagnosis in previous year** | - | 4 (0.5) | - | - | - | - |

-: Variables not collected at 6-month and/or 12-month follow-up.

* Variables collected in an additional survey implemented within the hospital routine
